# Malignant Throat Diseases

**Published:** 1875-02

**Authors:** 


### MALIGNANT THROAT DISEASES.

In these days, when diptheria is rife, almost all acute affections of the glands of the neck, the fauces, and the upper air-passages are taken for that disease. It is safe to say that not one sore throat in twenty is true diptheria. Patches of tough, false membrane, or of exudations which resemble it, may even exist, and the true diptheritic condition may still be absent. As good a test of the malignant quality of the attack as we can mention, is the exceedingly offensive odor which we have never known to fail in the genuine disease. Another is the great swelling or congestion of the surrounding parts. This enlargement is quite perceptible externally, and gives the impression that mumps or quinzy is present. When the terrible fetor and the great engorgement of the parts appear in combination, there need be no doubt as to the character of the disease.

The theory of the disease is that it is contagious, that is to say, that persons inhaling the breath or coming in direct contact with other emenations from the infected person, are quite likely to take the disease. It appears to be well established that it is at first purely local—its locality the throat and fauces—and that it is produced by a dormant organism, a parasite, called bacteria, which can only be developed by lodgement in the mucus membrane, and by a certain degree of temperature. These are believed to induce the false membrane by burrowing beneath the surface, and causing an exudation of peculiar, tenacious mucus, which presently becomes a stringy and leathery membrane. Ultimately, it is conceded, these minute organisms called bacteria reproduce themselves in great multitude, and are absorbed into the circulation. The disease thus becomes constitutional, and demands constitutional treatment.

If these theories are correct, two methods of treatment are clearly pointed out; the one local, the other constitutional. In another article we have stated the local means which we have successfully employed in many cases of greater or less malignity. So far as controlling the congestion, overcoming the fetor, reducing the swelling, inducing the loosening of the membranous deposit, and promptly increasing the breathing space in the upper air-passages, the ice-treatment has seemed, in our own experience, to leave nothing to be desired. Dr. Jackson, of Dansville, at whose institution a large number of these cases have been successfully treated, employs the ice precisely as we do, and with abundant success; but he supplements this treatment with steam inhalations, and obtains by this means the most gratifying results in the speedy softening of the tenacious mucus, and the rapid control of the local inflammation.

The common practice with the profession is to administer tonics, such as iron and quinine, for the purpose of sustaining the system, and to employ escharotics and astringents locally, to destroy and dislodge the bacteria, and to overcome the morbid condition. Anteseptics, such as chlorate of potash, are also largely relied upon, and are no doubt successful in many cases. But while this method is undoubtedly useful, it has not resulted as happily in our own experience as the ice-treatment, accompanied with the constitutional means which we will indicate. In fact, the chlorate of potash has not unfrequently induced a low, typhoid condition, with its attendant diarrhœa, although controlling the ulcerative processes with marked power.

Our constitutional treatment—aside from the diet of pure, fresh, sweet milk, which we insist upon—is mainly to see that the skin is kept active. The Dansville treatment accomplishes this by means of often-renewed wet-sheet packs, a most powerful and salutary method of equalizing the circulation, and reducing the febrile condition. This, or any other plan for inducing copious perspiration, may be employed. But we find perspiration to be of great importance, not only in lowering the excessive heat of the body, but also in physicing the system; in other words, in throwing off the disease gems through those sources of the waste and poison of the body—the perspiratory orifices. Abundance of good air, and copious baths of sunshine are essential. The windows MUST be kept open, so that the pure external air shall freely enter. Warmth may be kept up by adding clothing; never by shutting up the crevices and putting on weather strips. Few cases will terminate fatally if our system of treatment is followed.

---

SPONTANEOUS EPIDEMICS.—It has long been an important question as to where a disease comes from when it first makes its appearance in a locality? It is at length clearly established that all sorts of diseases may occur spontaneously, and appear as epidemics without being propagated by contagion, although it may be extended by contagion after the first outbreak. All known contagious diseases may be produced originally, under certain atmospheric and sanitary conditions, without contact with a diseased person. Small-pox, and even the plague, have arisen spontaneously, all over Europe and Asia, and these, as well as all contagious diseases, appear to possess the power of self-propagation under favorable conditions. By favorable conditions we of course mean conditions unfavorable to health. In other words, if cleanliness and good air abound, plague and pestilence will skulk away in search of a more favorable locality. In this view, the importance of pure air, pure sunlight, and pure skins will be evident.

